# Interaction between photosynthetic electron transport and chloroplast sinks triggers protection and signalling important for plant productivity

**DOI:** 10.1098/rstb.2016.0390

**Published:** 2017-08-14

**Authors:** Peter J. Gollan, Yugo Lima-Melo, Arjun Tiwari, Mikko Tikkanen, Eva-Mari Aro

**Affiliations:** Molecular Plant Biology, Department of Biochemistry, University of Turku, 20014 Turku, Finland

**Keywords:** photosynthesis regulation, PSI photoinhibition, chloroplast signalling, CO_2_ fixation, oxylipins

## Abstract

The photosynthetic light reactions provide energy that is consumed and stored in electron sinks, the products of photosynthesis. A balance between light reactions and electron consumption in the chloroplast is vital for plants, and is protected by several photosynthetic regulation mechanisms. Photosystem I (PSI) is particularly susceptible to photoinhibition when these factors become unbalanced, which can occur in low temperatures or in high light. In this study we used the *pgr5 Arabidopsis* mutant that lacks ΔpH-dependent regulation of photosynthetic electron transport as a model to study the consequences of PSI photoinhibition under high light. We found that PSI damage severely inhibits carbon fixation and starch accumulation, and attenuates enzymatic oxylipin synthesis and chloroplast regulation of nuclear gene expression after high light stress. This work shows that modifications to regulation of photosynthetic light reactions, which may be designed to improve yield in crop plants, can negatively impact metabolism and signalling, and thereby threaten plant growth and stress tolerance.

This article is part of the themed issue ‘Enhancing photosynthesis in crop plants: targets for improvement’.

## 1. Introduction

The pressing need to improve plant productivity has prompted a focus on increasing photosynthetic yield. One approach is to modify mechanisms that naturally downregulate photochemical efficiency [1–5], especially non-photochemical quenching (NPQ) of excitation from the major light-harvesting complex (LHCII), which protects photosystem II (PSII) during increases in light intensity [6,7]. Improving the rate of NPQ relaxation after a period of high light was recently shown to improve plant yield in fluctuating natural light by 15% [8]. Another avenue for improving photosynthetic yield is to increase the capacity for electron consumption in the chloroplast by strengthening transitory electron sinks in the chloroplast or permanent carbon sinks in specialized plant organs [1,2,8,9]. Strong sink demand not only improves growth and yield [10,11], but is important for tolerance to low temperature [12,13], where it is a factor in avoiding inhibition of photosystem I (PSI). Inhibition of electron consumption in the chloroplast, induced by low temperature, leads to accumulation of electrons in the photosynthetic electron transport chain, even in low light, causing formation of superoxide (O_2_^•–^) that specifically damages iron-sulfur (FeS) clusters in PSI centres [14–16]. The same mechanism can also cause PSI photoinhibition under high irradiance in the absence of low temperature stress [17]. Recovery from PSI photoinhibition involves the degradation and replacement of the entire PSI centre, which occurs over several days [18].

In this work, we addressed the role of proper regulation of photosynthetic electron transport reactions in the plant's response to a changing light environment. To this end we used an *Arabidopsis thaliana* mutant lacking the proton gradient regulation 5 (PGR5) protein, which is required for formation of a thylakoid membrane ΔpH under high light [19]. The molecular function of PGR5 has not been fully resolved, but the protein is commonly thought to be involved in the transport of electrons from PSI to plastoquinone (PQ) in one of two so-called ‘cyclic electron transport’ (CET) pathways (reviewed in [20]). The lack of lumen acidification means that during low-to-high light transitions the *pgr5* mutant can neither engage NPQ, nor control the transport of electrons from PQ to plastocyanin (PC) through the cytochrome *b_6_f* complex [19,21]. This means that during high light phases, the flow of electrons through the linear electron transfer pathway is unregulated in *pgr5*, leaving PSI highly exposed to over-reduction and photoinhibition [17,19,22]. The *npq4* mutant was included here as a control where NPQ is also missing, but the control of cytochrome *b_6_f* is retained [21,23]. Thus the difference between *pgr5* and *npq4* mainly concerns the regulation of electron flow via the cytochrome *b_6_f* complex, which is fully operational in *npq4* but missing from *pgr5* in high light (reviewed in [20]). We confirm that imbalanced accumulation of electrons in the electron transport chain rapidly induces PSI damage in *pgr5* [17,22] and demonstrate the broad and severe effects on primary and secondary metabolism, as well as on chloroplast signalling and nuclear gene expression. Deeper understanding of these processes is required to avoid unexpected fitness penalties, and is a key step in developing sustainable strategies for more efficient utilization of photosynthesis in crop plants.

## 2. Material and methods

### (a) Plants and growth conditions

*Arabidopsis thaliana* ecotypes Columbia-0 (Col-0) and Columbia *glabra 1* (*gl1*) were used as controls for *npq4* and *pgr5* mutants, respectively. Plants were grown for six weeks in a phytotron at 23°C, relative humidity 60%, 8 h photoperiod under constant white growth light (GL) of 120 µmol photons m^−2^ s^−1^. High light (HL) treatments involved shifting plants from GL to 1000 µmol photons m^−2^ s^−1^ in a temperature-controlled growth chamber set at 23°C.

### (b) Photochemistry and CO_2_ assimilation measurements

Photosystems II and I photochemical parameters were simultaneously measured using a Dual-PAM-100 system (Walz, Germany) based on chlorophyll *a* fluorescence [24] and the P700 oxidation signal [25] methods, respectively. Measurements of photochemical parameters were taken with a photosynthetic photon flux density (PPFD) gradient of five increasing steps (23, 54, 127, 431 and 1029 µmol photons m^−2^ s^−1^) measured in each leaf. Data were logged after 5 min from the start of each light intensity. CO_2_ assimilation was measured in leaves in 400 ppm or 2000 ppm CO_2_ at 23°C using the LI-6400XL Portable Infrared Gas Exchange System (LI-COR Biosciences, USA). Gas exchange parameters were taken with a PPFD gradient of eight increasing steps (0, 25, 50, 125, 300, 600, 1000 and 1600 µmol photons m^−2^ s^−1^) measured in each leaf. Data were logged after infrared gas analyser (IRGA) parameters reached a steady-state value after the start of each light intensity (usually around 120 s).

### (c) Starch quantification and electron microscopy

Starch content of leaves was measured using a total starch assay kit (Megazyme, Ireland) according to the accompanying protocol. From the same plants, the seventh leaf was harvested and fixed with glutaraldehyde for transmission electron microscopy (TEM) imaging at the Laboratory of Electron Microscopy at the University of Turku Medical Faculty, Turku, Finland.

### (d) RNA isolation and transcriptome analysis

Whole rosettes were treated with GL and HL for the time periods described, during the middle of the photoperiod. Immediately following treatment, leaves were detached and frozen in liquid N. Leaf samples contained at least four leaves from separate individual plants. Frozen leaves were ground in liquid N and total RNA was isolated using TRIsure (Bioline, USA) according to the protocol supplied, with an additional final purification in 2.5 M LiCl overnight at −20°C. Total RNA was used in RNAseq library construction. Libraries were sequenced in 50 bp single end reads using Illumina Hiseq 2500 technology (BGI Tech Solutions, Hong Kong). Reads were aligned to the reference genome build *Arabidopsis thaliana* TAIR 10 with Ensembl genes and transcripts annotation, using Strand NGS 2.7 software (Agilent, USA). Aligned reads were normalized and quantified using the DESeq R package. Gene expression fold changes were calculated using a two-way ANOVA test on triplicate samples (*n* = 3) with Benjamini–Hochberg *p*-value correction to determine the false discovery rate (FDR) for each gene. Significantly enriched Gene Ontology for Biological Process (GO-BP) terms were identified within gene lists using the enrichment analysis tool of the Gene Ontology Consortium (http://geneontology.org/).

### (e) 12-Oxo-phytodienoic acid measurements

Leaf tissues of plants were harvested and immediately frozen in liquid N. Ground samples were extracted in methanol, and metabolites were separated and detected by UPLC-MS. 12-Oxo-phytodienoic acid (OPDA) abundance was quantified relative to fresh weight in five samples (*n* ≥ 3).

### (f) Lipid peroxidation imaging and quantification

Lipid peroxidation was assessed by visualizing auto-luminescence *in planta* [26]. After light treatment, rosettes were incubated in darkness for 2 h, before the luminescence signal was collected for 20 min using an IVIS Lumina II system (Caliper Life Sciences, USA) containing an electrically cooled CCD camera.

### (g) Singlet oxygen quantification with electron paramagnetic resonance

Singlet oxygen trapping was performed in isolated thylakoids from GL- and HL-treated WT and *pgr5* plants as described in [27] using a Miniscope (MS5000) electron paramagnetic resonance (EPR)-spectrometer equipped with a variable temperature controller (TC-HO4) and Hamamatsu light source (LC8). The isolated thylakoids equivalent to 150 µg ml^−1^ chlorophyll were illuminated under actinic light (2000 µmol photons m^−2^ s^−1^) for 180 s in the presence of vacuum distilled 2,2,6,6-tetramethylpiperidine (TEMP) (50 mM). Subsequently, the samples were centrifuged at 6500***g*** for 3 min and the supernatant was used for EPR measurements. The measurements were conducted at frequency 9.41 GHz, centre field 3363 G, field sweep 150 G, microwave power 3 mW and modulation frequency 100 kHz with modulation width of 2 G. The final spectra were obtained by three accumulations of each sample.

## 3. Results

### (a) High light treatments induce different malfunctions in photosynthetic light reactions in *pgr5* and *npq4*

**Figure 1.**
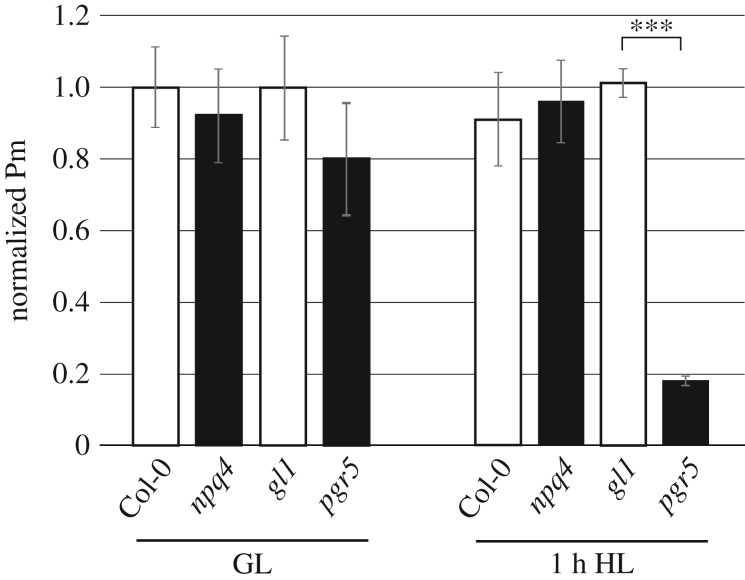
Functional PSI content in Col-0, *gl1*, *pgr5* and *npq4* plants previously treated with growth light (GL) or high light (HL). The maximum amount of oxidizable P700 (Pm) was determined using 5 s far-red irradiation followed by a saturating pulse of actinic light. Pm values are shown normalized to the respective WT GL sample. Error bars show standard deviation among replicates (*n* = 4). Asterisks represent significant differences between *pgr5* and *gl1* within the same light treatment (Student's *T* test, *p* < 0.001).

**Figure 2.**
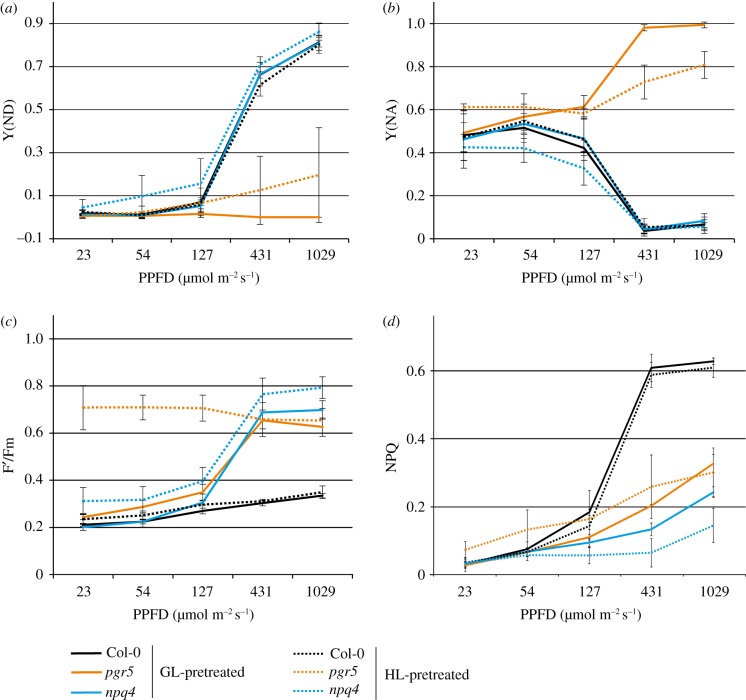
Analysis of PSI and PSII function under increasing light intensities by chlorophyll *a* fluorescence and P700 oxidation, in Col-0, *pgr5* and *npq4* plants pretreated with growth light (GL) or high light (HL). (*a*) Limitation of electron transfer to the donor (lumenal) side of PSI; (*b*) limitation of electron transfer from the acceptor (stromal) side of PSI; (*c*) the operational state of PSII reaction centres, which are open (active) at low F′/Fm values and closed (inactive) at high F′/Fm values; (*d*) non-photochemical quenching (1 − (Fm′/Fm)). Error bars show standard deviation among replicates (*n* = 4).

In order to separate the effects of cytochrome *b_6_f* regulation from NPQ, we compared the *Arabidopsis pgr5* and *npq4* mutants, and their respective WTs *gl1* and Col-0. PSI function was determined using the maximum oxidation capacity of P700 at the PSI reaction centre (Pm), measured in parallel in plants that were previously treated for 1 h with either 120 µmol photons m^−2^ s^−1^ (GL) or 1000 µmol photons m^−2^ s^−1^ (HL). The Pm value in *pgr5* plants from GL was lower than in the other genotypes, although this difference was not statistically significant (figure 1). In *npq4* and both WTs the Pm was not affected by the 1 h HL treatment (figure 1). However HL treatment led to a severe decrease of Pm in *pgr5*, to around 25% of its GL level, as previously reported [17,19,22]. PSI donor side limitation was rapidly induced in *npq4* and both WTs in measurements where light intensities were higher than GL, irrespective of the previous light treatments, which corresponded with an equivalent decline in acceptor side limitation (figure 2*a*,*b*). Induction of PSI donor side limitation was completely missing from the *pgr5* mutant, whereas strong acceptor side limitation occurred in *pgr5* plants in light intensities above GL, which demonstrated excess electron transport in relation to stromal electron acceptors [21,28]. The *pgr5* mutants treated with HL for 1 h showed lower acceptor side limitation at higher light intensities, which is likely due to HL-induced PSI damage that decreased electron transport to the stromal acceptors.

The operational state of PSII was assessed using the fluorescence parameters F′, which is the fluorescence of chlorophyll *a* under actinic light, and Fm, which is the maximum chlorophyll *a* fluorescence. The F′/Fm calculation was used in preference to routine Fv/Fm calculations to avoid the confounding effect of PSI damage that is a critical factor in *pgr5* analysis [22,29]. In both GL- and HL-treated WT leaves, low F′/Fm values over increasing light intensity showed that PSII remained open (figure 2*c*). On the contrary, increases in F′/Fm occurred in GL-treated *pgr5*, and in both GL- and HL-treated *npq4* leaves, demonstrating an increase in the number of closed PSII reaction centres in the mutants at light intensities above GL (120 µmol photons m^−2^ s^−1^). This can be attributed to the lack of NPQ under high light, which is shown in figure 2*d* to increase sharply at higher light intensities in WT, but not in the two mutants under the above-mentioned conditions. HL-treated *pgr5* plants behaved differently, demonstrating high F′/Fm at low light intensities. This may be due to PSI damage incurred during the 1 h HL treatment that limited PSI activity and caused over-reduction of the electron transport chain [17,30], leading to PSII closure in low light. The small decrease in F′/Fm in HL-treated *pgr5* leaves at high irradiance suggests that PSI damage may limit electron transfer in low light more than in high light.

### (b) Photosystem I damage has direct consequences for stromal metabolism

**Figure 3.**
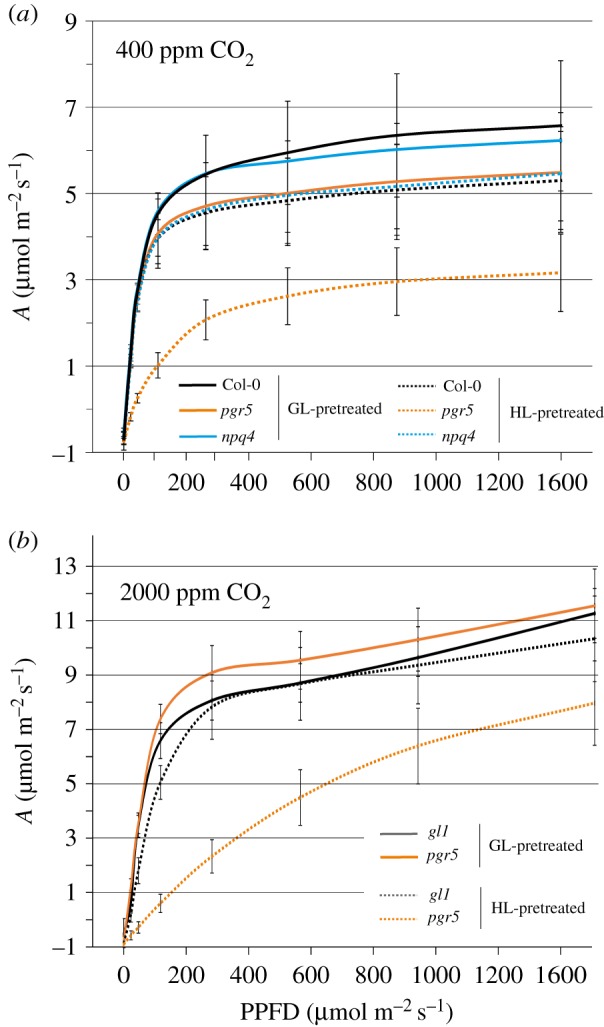
(*a*) Light curves of CO_2_ fixation in Col-0, *pgr5* and *npq4* leaves pretreated with growth (GL), or high light (HL) at 400 ppm CO_2_; (*b*) light curves of CO_2_ fixation in *gl1* and *pgr5* leaves pretreated with growth (GL), or high light (HL) at 2000 ppm CO_2_.

In order to further assess the effects of the observed PSI damage on primary stromal metabolism in different light intensities, we first monitored the light curves of CO_2_ fixation in WT, *pgr5* and *npq4* plants treated beforehand with GL and HL, as described in §3a above, under ambient CO_2_ concentration (400 ppm). Light limitation of photosynthesis, as determined by the steepest part of each light curve of CO_2_ fixation, occurred until a PPFD of approximately 120 µmol m^−2^ s^−1^ in all GL-treated plants and in HL-treated WT and *npq4* plants (figure 3*a*). Light saturation of CO_2_ fixation above PPFD of 120 µmol m^−2^ s^−1^ demonstrated a shift to CO_2_ as the limiting factor for photosynthesis. HL treatment caused a small decrease in the maximum level of CO_2_ fixation under high PPFD in WT and *npq4* that was approximately the same as the level in GL-treated *pgr5*. In sharp contrast to the other plants, HL-treated *pgr5* showed much lower CO_2_ fixation under low light intensities, with the maximum CO_2_ fixation rate reduced to approximately 60% of GL levels. The shift from light limitation to CO_2_ limitation in HL-treated *pgr5* occurred at a PPFD of around 400 µmol photons m^−2^ s^−1^.

The light response curves of CO_2_ fixation were repeated under high CO_2_ concentration (2000 ppm) for the *pgr5* and WT plants that had been GL- and HL-treated exactly as before. Here, CO_2_ fixation at high PPFD in both GL- and HL-treated *pgr5* was 2–2.5 fold higher compared to ambient CO_2_. The level of CO_2_ fixation in GL-treated *pgr5* was slightly elevated in comparison to GL- and HL-treated WT, while HL-treatment of *pgr5* reduced CO_2_ fixation at high PPFD to around 70% of that measured for GL-treated plants (figure 3*b*). The light response curve of HL-treated *pgr5* at high CO_2_ did not achieve a steady rate of CO_2_ fixation within the PPFD range used, which shows that photosynthesis was not limited by CO_2_ availability.

**Figure 4.**
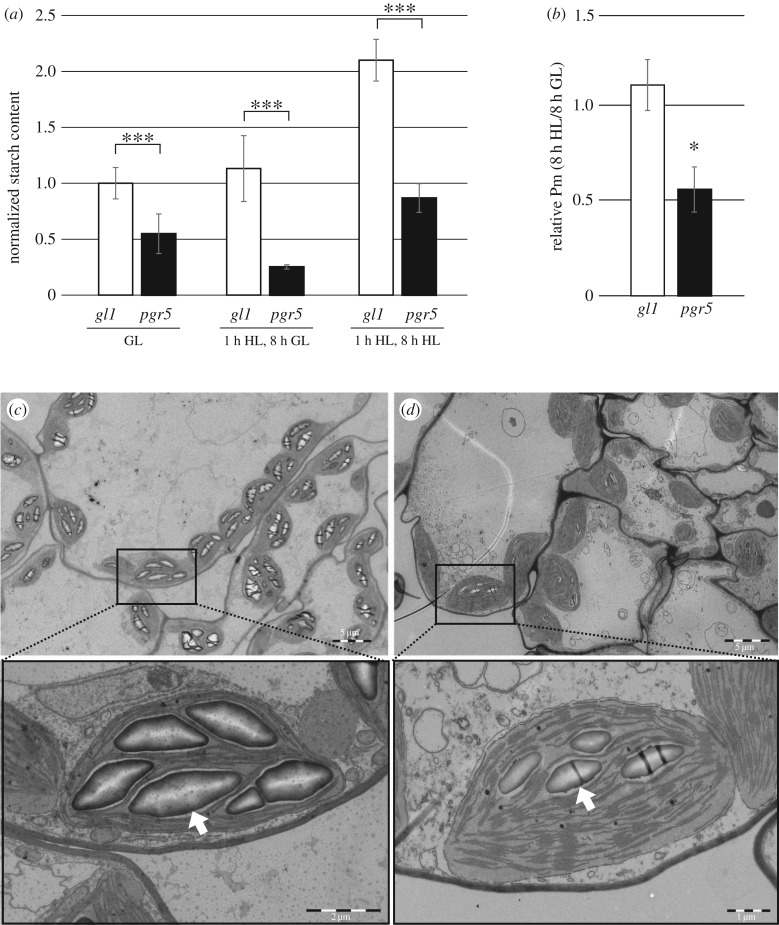
Starch accumulation in WT and *pgr5* plants treated with growth light (GL) or with high light (HL). (*a*) Plants were taken from GL, or treated with 1 h HL and then exposed to either regular growth conditions (8 h GL) or HL (8 h HL) during the following day. Samples were harvested at the end of the following day. Starch abundances were calculated as percentages of fresh weight and are shown normalized to the *gl1* GL sample. Error bars show standard deviation among replicates (*n* = 3). (*b*) Pm in *pgr5* and WT treated with 1 h HL and then 8 h HL, relative to Pm after 8 h GL in the same genotype; asterisks represent significant differences between *gl1* and *pgr5* (*Student's *t* test *p* < 0.05; ***Student's *t* test *p* < 0.001); (*c*,*d*) Transmission electron micrographs demonstrating the sizes and abundance of starch granules in leaf 7 of 1 h HL, 8 h GL treated WT (*c*) and *pgr5* (*d*) chloroplasts. Lower panels show high resolution views of selected areas of upper panels. White arrows indicate representative starch granules. Scale bars show size.

To determine the effect of PSI damage at the chloroplast metabolic level, starch content was investigated in WT and *pgr5* mutants that were subjected to the GL and HL treatments described above and then shifted to regular growth conditions until the end of the following day to allow diurnal starch accumulation. The starch contents of *pgr5* leaves treated with GL or HL were 50% and 25%, respectively, of WT levels under the same conditions. HL treatment approximately halved the starch content in *pgr5* compared to GL treatment (figure 4*a*). Another set of plants were HL-treated for 1 h and then, instead of transferring to GL, were exposed to the same intensity of HL throughout the following day. These plants showed increases in starch content of around 100% for WT, and 350% for *pgr5*, in comparison to HL-treated plants that were shifted to GL (figure 4*a*). These increases in starch accumulation after 8 h in HL occurred alongside no change to Pm in WT plants, but a 50% reduction in Pm in *pgr5* plants, in comparison to 8 h in GL (figure 4*b*). Chloroplast ultrastructure (transmission electron micrographs) clearly showed the smaller size and lower abundance of accumulated starch granules in *pgr5* that had been treated with HL on the previous day, in comparison to WT leaves (figure 4*c*,*d*). The lower starch content in GL-treated *pgr5* compared to WT, as measured in the assay (figure 4*a*), was not evident from transmission electron micrographs (not shown).

### (c) The transcription profiles of *pgr5* and *npq4* mutants are altered during light stress and recovery

The transcriptomes of *pgr5* mutants from GL, after 1 h of HL, and after 1 h of recovery in GL following HL treatment, were analysed to investigate the impact of thylakoid ΔpH on nuclear gene expression under changes in light intensity. Transcriptomes of the *npq4* mutant were analysed in parallel to identify transcriptional changes that in *pgr5* may be attributed to missing NPQ. Global effects of the *pgr5* mutation on gene expression were identified as enriched Gene Ontology (GO) terms within lists of significantly differentially-regulated genes. Of the six groups that were analysed (up- and downregulated genes from each condition), only the downregulated genes in 1 h HL and 1 h GL recovery contained statistically significantly enriched GO terms (table 1). In both cases, the term ‘jasmonic acid metabolic process' (GO:0009694) was the most highly enriched at around 12-fold, while other jasmonate-related signalling processes were also significantly enriched. Responses to HL (GO:0009644), hydrogen peroxide (H_2_O_2_; GL:0042542), salicylic acid (GO:0009751) and ethylene (GO:0009723) were also found to be enriched in downregulated genes in *pgr5* after HL stress and/or after 1 h recovery in GL (table 1).

**Table 1.** Significantly enriched Gene Ontology Biological Process (GO-BP) terms in lists of genes differentially expressed in *pgr5* mutants.

GO term	description	included genes (total genes)^a^	fold enrichment	*p*-value (Bonferroni corrected)
enriched terms in downregulated genes; *pgr5* 1 h HL/*gl1* 1 h HL				
GO:0009694	jasmonic acid metabolic process	10 (27)	12.95	2.14 × 10^−5^
GO:0009611	response to wounding	38 (184)	7.22	3.18 × 10^−17^
GO:0009867	jasmonic acid-mediated signalling	12 (61)	6.88	6.84 × 10^−4^
GO:0042542	response to hydrogen peroxide	10 (53)	6.60	9.38 × 10^−3^
GO:0009644	response to high light intensity	11 (60)	6.41	4.13 × 10^−3^
GO:0009751	response to salicylic acid	20 (171)	4.09	4.64 × 10^−4^
GO:0009723	response to ethylene	27 (235)	4.02	5.33 × 10^−6^
enriched terms in downregulated genes; *pgr5* 1 h GL recovery/*gl1* 1 h GL recovery				
GO:0009694	jasmonic acid metabolic process	12 (27)	11.77	2.14 × 10^−6^
GO:0006568	tryptophan metabolic process	8 (24)	8.83	1.08 × 10^−2^
GO:0009753	response to jasmonic acid	38 (178)	5.65	1.06 × 10^−13^
GO:0009611	response to wounding	34 (184)	4.89	2.63 × 10^−10^
GO:0009751	response to salicylic acid	27 (171)	4.18	2.49 × 10^−6^

^a^Number of genes under each GO-BP term that were present in the *pgr5* differentially-expressed gene list, and total number of genes in the GO-BP term are shown in parentheses.

The expression of individual genes undergoing significant fold change (FC) were investigated in further detail. The genes encoding enzymes involved in biosynthesis of OPDA, the chloroplast precursor for the hormone jasmonic acid (JA), were strikingly downregulated in *pgr5* plants compared to WT under HL stress and during recovery. This included chloroplast lipid peroxidases, allene oxide synthase and cyclases, as well as the chloroplast lipase DAD1, OPDA reductase and numerous JA signalling regulation (JAZ) intermediates (table 2). In WT, oxylipin synthesis enzymes were significantly upregulated by HL and, in general, further upregulated during recovery (see electronic supplementary material, file S1); however, this did not occur in *pgr5*, which is seen as significant downregulation in HL and recovery compared to WT in most cases (table 2).

**Table 2.** Distinctive differentially expressed genes in *pgr5* and *npq4 Arabidopsis* before and after high light stress (GL and 1 h HL, respectively) and during recovery (1 h GL). Numbers show log2-fold change in expression in *pgr5* and *npq4* mutants in comparison to the respective WT samples under identical treatments. Yellow indicates genes with ≥log2 (1) expression (significantly upregulated) and blue indicates genes with ≤log2 (−1) expression (significantly downregulated).

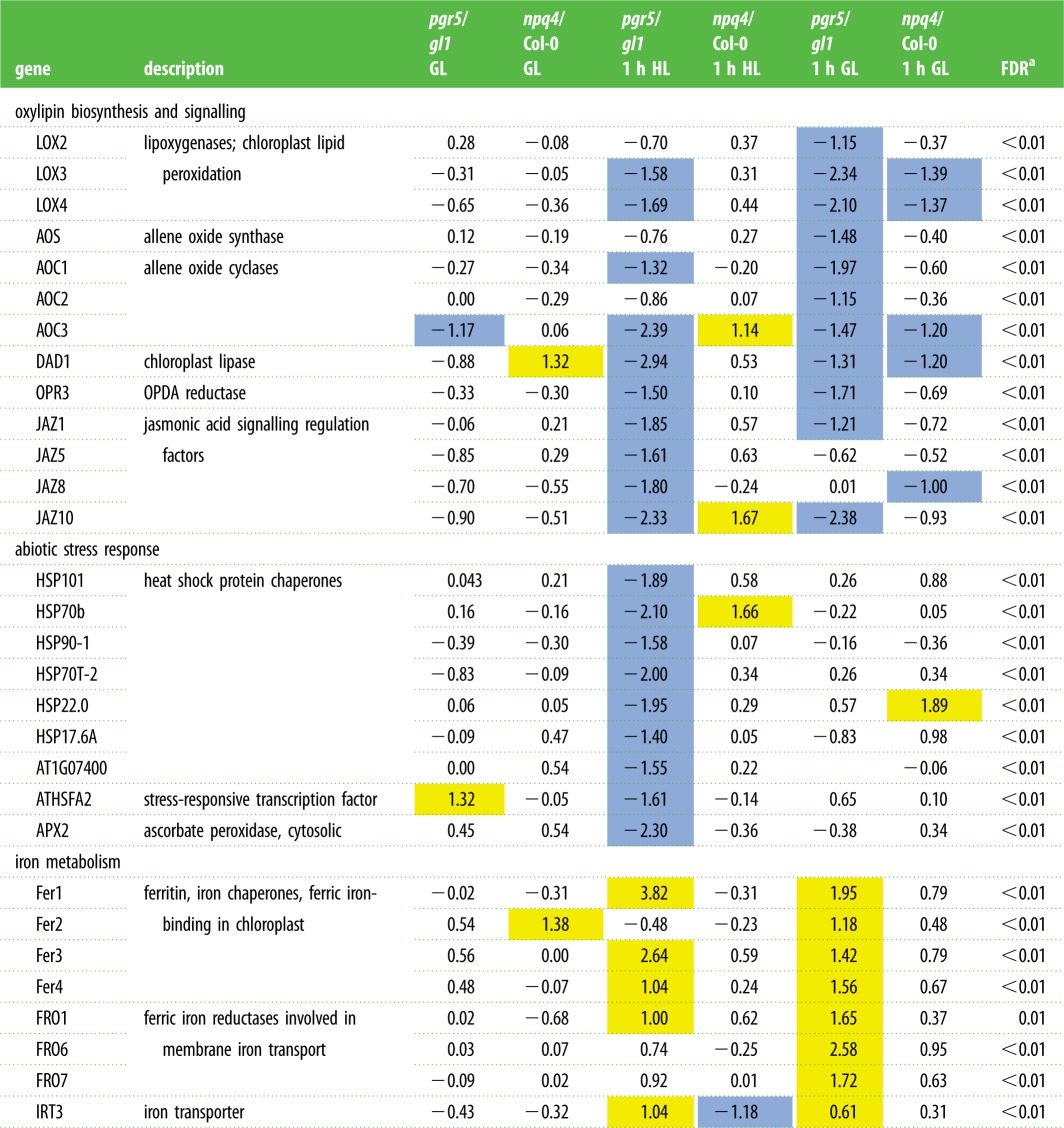

^a^False discovery rate calculated using the Benjamini–Hochberg procedure.

**Figure 5.**
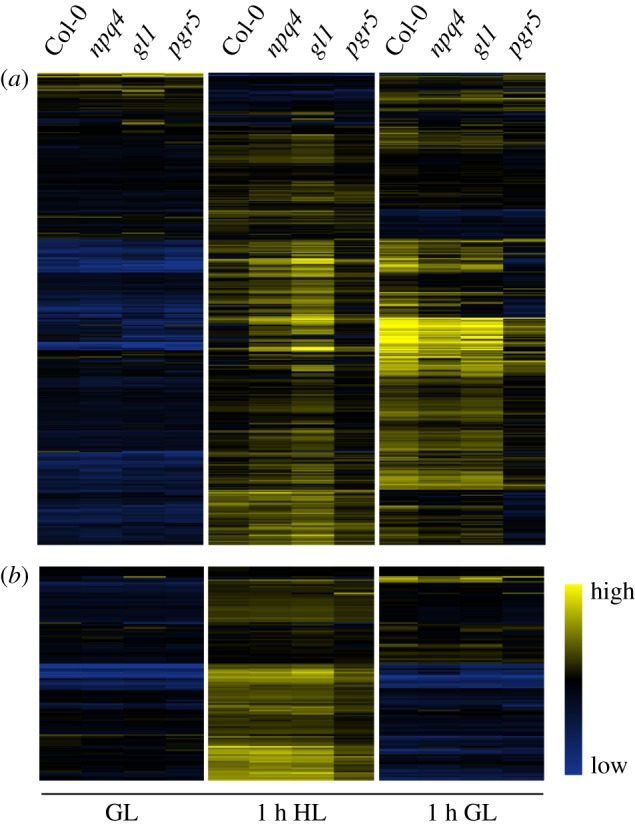
Clustered heatmap of high light-responsive genes in Col-0, *npq4*, *gl1*, and *pgr5* leaves before (GL) and after (1 h HL) high light treatments, or during recovery (1 h GL): (*a*) approximately 400 genes induced by 12-oxophytodienoic acid (OPDA) were downregulated in *pgr5* compared to *gl1* in 1 h HL and 1 h GL treatments (see text for details); (*b*) H_2_O_2_-responsive genes (GO:00423542) were upregulated by 1 h HL treatment, but were under-expressed in *pgr5* compared to the other genotypes. Clustered heatmap shows the absolute expression of each gene in Col-0, *gl1*, *npq4* and *pgr5* under each light treatment. Legend shows colours that represent high, intermediate and low expression.

Based on the observed under-expression of genes involved in OPDA and oxylipin synthesis pathways in *pgr5,* relative to WT, the effect of light-induced OPDA signalling on nuclear gene expression was investigated in *pgr5* and *npq4* mutants. The expression of about 400 genes that were previously shown to be upregulated in response to OPDA treatment [31] was analysed in the current transcriptomics data. In all genotypes, these genes were expressed at relatively low levels in the original GL and were upregulated by HL treatment. In *npq4* and both WT plants, a large proportion of OPDA-induced genes was further upregulated during the recovery period (figure 5*a*). In contrast, most of these genes were under-expressed in *pgr5* in comparison to its WT (*gl1*) after 1 h HL, and after recovery for 1 h at GL (see electronic supplementary material, file S2 for transcription details).

The expression profiles of the 130 most strongly attenuated genes in *pgr5* were analysed in publicly-available expression data using the Genevestigator database [32]. Strong upregulation of this gene set was identified in HL and drought stresses and treatments with OPDA and methyl jasmonate, and also by infection with many biotic stresses including bacterial, fungal and herbivorous pathogens (electronic supplementary material, figure S1). The same gene set was considerably, but not entirely, downregulated in darkness, in iron deficiency, and in mutant plants with interrupted PSI function (*psad1-1* and *psae1-3*), and in mutants lacking the JA signalling intermediate coronatine insensitive 1 (*coi1*).

High light stress is well known to upregulate the so-called ‘heat shock protein’ (HSP) chaperones involved in abiotic stress response [33,34]. HSP gene transcription in *pgr5* was highly upregulated in HL (15–1000 FC) and subsequently downregulated during recovery, in a trend similar to WT (see electronic supplementary material, file S1). However, many HSPs and other heat shock factors were significantly less upregulated in *pgr5* in HL compared to WT (table 2), suggesting under-production of an abiotic stress signal in *pgr5* during HL. Expression of many abiotic stress-responsive genes is linked to H_2_O_2_ signalling [35,36], and so the expression of genes included in the GO term ‘response to H_2_O_2_’ (GO:0042542) was assessed in our RNAseq data. Strong upregulation of these genes under HL was evident in all genotypes, but was clearly lower in *pgr5* than in *npq4* and the WT plants (figure 5*b*). To investigate whether this may be due to increase in reactive oxygen species (ROS) scavenging in *pgr5,* the expression of almost 100 enzymes responsible for dealing with oxidative stress was assessed, including many superoxide dismutases, catalases and peroxidases. Among these genes, only the cytosolic ascorbate peroxidase (APX2) was significantly differentially-expressed in *pgr5* (table 2). Although strongly upregulated under HL in both *pgr5* (30 FC from GL) and WT (200 FC from GL), APX2 was markedly under-expressed in *pgr5* compared to WT.

Genes involved in iron metabolism, including several chloroplast ferritin (Fer) iron chaperones and ferric iron reductase (FRO) enzymes, were significantly upregulated in *pgr5* during and/or following HL stress, in comparison to its WT (table 2). In fact, Fer1 and Fer3 genes were both upregulated in all genotypes by HL stress in comparison to GL; however, the FC in *pgr5* (24 FC and 15 FC, respectively) was much greater than in WT (1.7 FC and 3.7 FC, respectively). FRO genes were downregulated by HL in all genotypes, but were strongly upregulated in *pgr5* during recovery.

### (d) Light stress induces synthesis of chloroplast oxylipins in WT, *pgr5* and *npq4*

**Figure 6.**
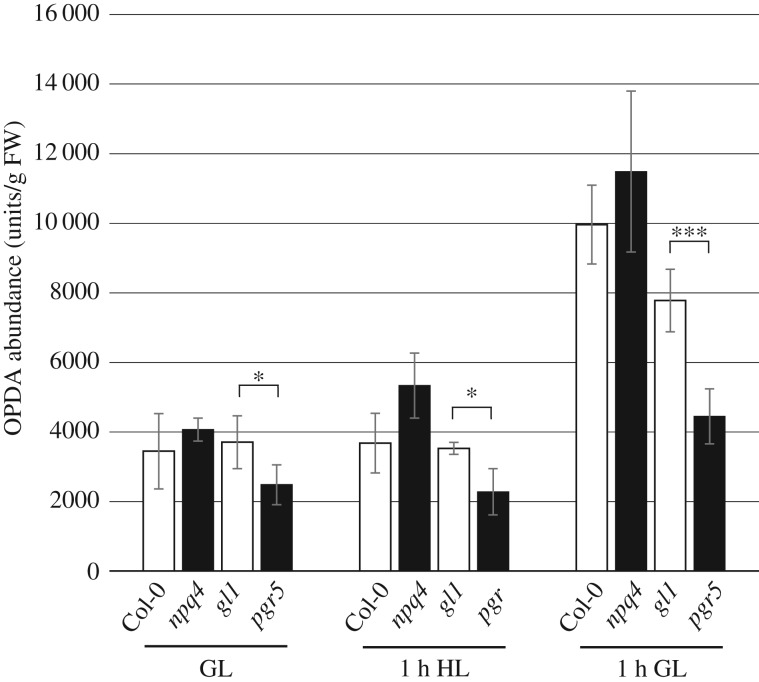
Abundance of OPDA in Col-0, *npq4, gl1* and *pgr5* leaves before (GL) and after 1 h HL treatments, and during recovery (1 h GL). Concentrations are expressed as peak area from mass spectrometry chromatograms/fresh weight. Error bars indicate standard deviation among replicate samples (*n* ≥ 3). Asterisks represent significant differences between *gl1* and *pgr5* in GL and 1 h HL (*Student's *t* test *p* < 0.05) and 1 h GL (***Student's *t* test *p* < 0.001).

We next analysed and compared the abundance of OPDA in *pgr5, npq4* and the WT plants treated with the same high light stress and recovery regimes described in §3c. This analysis demonstrated an increase in OPDA abundance after 1 h recovery in GL in all genotypes (figure 6). OPDA levels in *pgr5* were significantly lower than the WT in original GL conditions and after 1 h HL (*p* < 0.05), as well as after 1 h recovery (*p* < 0.001).

**Figure 7.**
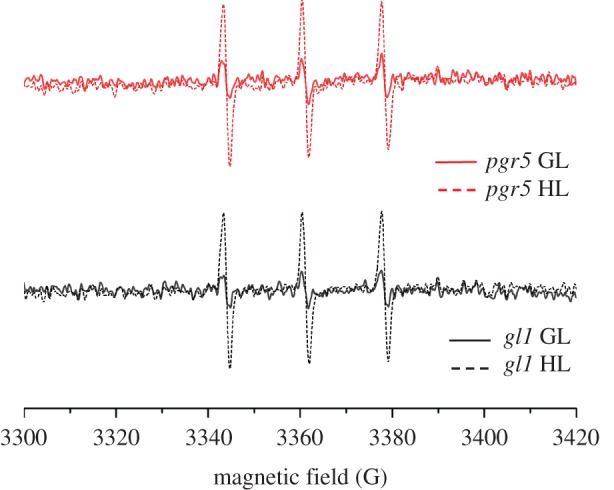
Singlet oxygen production in *gl1* and *pgr5* thylakoids pretreated with growth light (GL) or high light (HL). Traces show electron paramagnetic resonance (EPR) spectra with peaks indicating oxidized TEMP spin trap in the presence of purified thylakoids isolated from plants pretreated with 1 h GL (solid traces) or HL (dashed traces).

The synthesis and signalling of oxylipins in *Arabidopsis* has been linked to the generation of singlet oxygen (^1^O_2_) in PSII reaction centres [37,38]. Considering lower OPDA abundance and downregulation of OPDA-regulated genes in *pgr5*, we investigated the production of ^1^O_2_ in thylakoids with EPR, using an ^1^O_2_-specific spin probe. Thylakoids isolated from HL-treated plants produced higher amounts of ^1^O_2_ under saturating light than those isolated from GL-treated plants; however, the intensity of the ^1^O_2_ signal was indistinguishable between *pgr5* and WT thylakoids, indicating equivalent production of ^1^O_2_ in both genotypes (figure 7). HL-induced lipid peroxidation was qualitatively assessed *in planta* using a super-cooled CCD camera to image the native luminescence emitted by lipid peroxides [26]. This assay could not distinguish any differences in the level of lipid peroxidation between *pgr5* and WT after 1 h HL treatment (electronic supplementary material, figure S2).

## 4. Discussion

### (a) Direct interaction between photosynthetic electron transport and stromal metabolism

**Figure 8.**
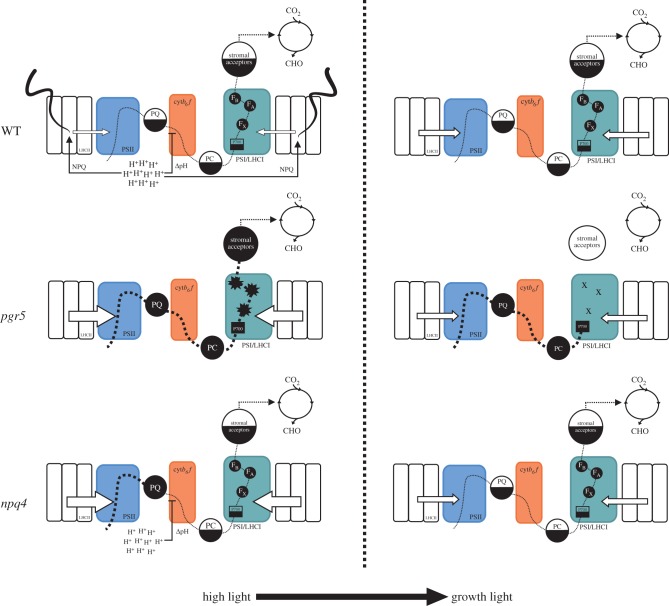
The consequences of distinct high light (HL) responses in WT, *pgr5* and *npq4* plants. In WT, HL causes lumen protonation that induces NPQ, which dissipates light-harvesting complex II (LHCII) excitation and maintains the plastoquinone (PQ) pool in a partially oxidized form. Lumen protonation also forms a thylakoid membrane proton gradient that slows electron transport through cytochrome *b*_*6*_*f*, which maintains partially oxidized forms of the plastocyanin (PC) pool, P700 and the stromal electron acceptors. After a HL phase, lumen protonation, NPQ and cytochrome *b_6_f* control are relaxed. The *pgr5* mutant lacks lumen protonation in HL, and therefore both NPQ and cytochrome *b_6_f* control are impeded. PQ, PC, P700 and stromal electron acceptors become saturated and excess electrons move to O_2_, forming ROS that inactivate F_A_, F_B_ and F_X_ iron-sulphur clusters in PSI. After the HL phase, photoinhibited PSI is unable to transport electrons from P700, causing reduction of electron carriers of photosynthetic light reactions and oxidation of electron acceptors in the chloroplast stroma. This downregulates CO_2_ reduction, which may contribute to decreases in starch accumulation and enzymatic oxylipin production. The *npq4* mutant lacks the PsbS protein, and therefore NPQ is absent under HL. The PQ pool can become over-reduced in HL; however, the partially oxidized states of PC, P700 and stromal acceptors are still maintained by lumen protonation and cytochrome *b_6_f* control in *npq4*. After a HL phase, lumen protonation and cytochrome *b_6_f* control are relaxed and the electron transport chain operates normally.

Sudden increases in light intensity generate increased electron current through the photosynthetic system, which is suppressed by activation of NPQ and downregulation of electron flow through the cytochrome *b_6_f* complex (reviewed in [39]). Both mechanisms depend on acidification of the thylakoid lumen, and both are affected in the *pgr5* mutant under HL [19]. Subsequently, increases in light intensity create in *pgr5* an over-supply of electrons from the light reactions, relative to the electron-accepting capacity of the stroma, leading to acceptor-side limitation at PSI. Electrons then move to the alternative electron acceptor oxygen, creating ROS that damage the PSI FeS clusters and inactivate PSI [14,17]. HL treatment of *pgr5* plants for 1 h drastically decreased the concentration of operational PSI centres (figure 1). This is in agreement with previous studies that have also showed PSI inactivation to be induced in *pgr5* by increases in light intensity [17,19,22,40]. In this work we exploited HL-inducible PSI photoinhibition in *pgr5* to study the ensuing effects of PSI damage on metabolic processes in the chloroplast (figure 8).

In the current work, CO_2_ fixation and starch accumulation were shown to be lower in *pgr5* compared to the WT, independent of light stress, while 1 h HL treatment of *pgr5* led to severe decreases in both traits (figures 3 and 4). A simple explanation for diminished primary and secondary metabolism in *pgr5* is the affected PSI electron transport, which is decreased in *pgr5* under GL [17] and severely inhibited by HL treatment (figure 1; [17,40]). Downregulated PSI activity would be expected to cause an under-supply of reducing power to the stroma, limiting metabolic reactions in *pgr5* chloroplasts, particularly after the HL exposure. Considering the role proposed for the PGR5 protein in CET, it may be argued that the observed decrease in stromal metabolism was due to limited ATP production in the *pgr5* mutant, and that PSI damage occurred through acceptor-side limitation caused by a low ATP : NADPH ratio [20,41]. However, we found that CO_2_ fixation in GL-treated *pgr5* plants under 2000 ppm CO_2_ was equivalent to the WT, and approximately double that measured at 400 ppm CO_2_ (figure 3*b*), which rules out the possibility of ATP limitation of the Calvin–Benson–Bassham (CBB) cycle in *pgr5.* This result is in agreement with the CO_2_ fixation rates in *PGR5-*knockdown rice lines that were similar to WT at both ambient CO_2_ and high CO_2_ [42]. A clear contradiction, however, appears between our results and a previous demonstration of inhibited CO_2_ fixation in the *Arabidopsis pgr5* mutant at high CO_2_ that was attributed to ATP deficiency [40]. This discrepancy may be partly due to the experimental set-up of the latter study, where plants were subjected to a six fold increase in light intensity for several minutes during the gas exchange analysis. This would have caused a degree of PSI photoinhibition in *pgr5*, which occurs very quickly during sudden increases in light intensities, as the authors pointed out [40]. Our light response curves of CO_2_ fixation were designed to minimize PSI damage by exposing plants to only 2–3 min at each PPFD, and by applying an ascending order of light intensities.

HL-induced damage to PSI in *pgr5* was especially deleterious to CO_2_ fixation under subsequent low light intensities, but this effect could be partially overcome by increasing the PPFD (figure 3). The high level of PSII closure in HL-treated plants under low PPFD (figure 2*c*) indicates that inhibited PSI activity causes over-reduction of electron carriers in the photosystem under low light phases (figure 8). Meanwhile high light intensities appear to more effectively excite the remaining functional PSI centres to improve CO_2_ fixation (figure 3), causing a small decrease in PSII closure (figure 2*c*). Higher *per capita* PSI activity under HL would also explain the marked improvement in starch accumulation in HL-treated *pgr5* plants that were subsequently exposed to 8 h at 1000 µmol photons m^−2^ s^−1^, despite 50% lower PSI activity, compared to those returned to GL for 8 h (figure 4). Such a scenario shows the importance of PSI protection under fluctuating light in order to maintain stromal metabolism, as highlighted in the devastating effect of fluctuating light on plants lacking PGR5 function [22,43].

### (b) Photosystem I damage attenuates chloroplast signalling

Redox imbalance within the photosynthetic electron transport chain impacts the cell through retrograde signalling that modifies nuclear gene expression (reviewed in [44]). In this work we sought to understand how PSI damage affects gene expression and chloroplast signalling. We found oxylipin signalling to be the most severely affected pathway of expression regulation in the *pgr5* mutant. In the WT, HL led to strong upregulation of hundreds of transcripts known to respond to the oxylipin hormone OPDA [31], which duplicates the light-sensitivity of OPDA synthesis and signalling that has been reported previously [45–48]. These transcripts were also upregulated by HL in *pgr5* in comparison to the GL levels (figure 5*a*), but were dramatically under-expressed in the mutant after HL stress and after 1 h recovery in GL, compared to WT (table 2 and figure 5*a*). This transcription phenomenon is in line with the production of only 55–70% of WT levels of OPDA in the *pgr5* under the conditions tested here (figure 6), and with the downregulated expression (relative to WT) of enzymes required for synthesis of OPDA (table 2). The opposite trend was evident in the *npq4* mutant, wherein OPDA (figure 6) and OPDA-sensitive transcripts (table 2 and figure 5*a*) were more abundant than in WT after the HL treatment, as expected [49,50]. Notably, upregulation of OPDA-sensitive genes was apparent after 1 h HL stress in all genotypes, whereas significant increases in OPDA abundance from GL levels were only apparent after 1 h recovery in GL. This may demonstrate the potency of OPDA as a transcription regulator, with undetected increases having a strong effect on expression induction.

Transcription of the genes encoding oxylipin enzymes is induced by OPDA [29], meaning that OPDA synthesis is auto-upregulated. This phenomenon can account for the large increases in OPDA concentration in all genotypes after 1 h recovery, i.e. the latest time-point (figure 6). The *pgr5* mutant had significantly lower OPDA concentrations than WT under all conditions analysed, prompting us to investigate factors upstream of OPDA biosynthesis in an attempt to delineate the cause and effect of low OPDA hormone and attenuated OPDA signalling in *pgr5*. Singlet oxygen (^1^O_2_), produced in the PSII reaction centre, is associated with upregulated expression of genes encoding oxylipin enzymes in *Arabidopsis* [38,51,52]. Accordingly, increased ^1^O_2_ production in the *npq4* mutant [53] corroborates the upregulation of enzymatic oxylipin production in *npq4* observed here and elsewhere [45,49,50]. Furthermore, a minor increase in ^1^O_2_ previously shown in chloroplasts treated with nigericin was attributed to the abolition of NPQ [53]. Since nigericin mimics the *pgr5* lesion by demolishing thylakoid ΔpH, we expected enhanced ^1^O_2_ production in *pgr5* mutants in HL; however, we found no difference between *pgr5* and WT in ^1^O_2_ production. The fact that our EPR measurements were performed on isolated thylakoids wherein NPQ could not be engaged might explain why *pgr5* did not produce more ^1^O_2_ than WT, but this result also indicates that OPDA downregulation in *pgr5* is not due to any *under-production* of ^1^O_2_ from PSII in HL, nor to a deficiency in lipid peroxidation (electronic supplementary material, figure S2) that provides the material for oxylipin production in the chloroplast [54,55]. The most likely explanation is downregulation of chloroplast metabolism as a result of decreased PSI activity in *pgr5*. In support of this, the expression of OPDA-responsive genes is also downregulated in *Arabidopsis* mutants with inhibited PSI function (*psad1-1*, *psae1-3*; electronic supplementary material, figure S1), and in the *stn7* mutant which has decreased excitation of PSI [48].

PSI photoinhibition in HL-treated *pgr5* (figure 8) is also a likely justification for the strong upregulation of several ferritin chaperones and iron reductase enzymes (table 2). Ferritin expression is upregulated in response to excess iron, to mitigate oxidative stress through iron chelation [56]. In HL, especially in the *pgr5* mutant, sequestration and mobilization of iron may be particularly important for efficient turnover of damaged PSI and to avoid Fenton's reaction with H_2_O_2_ that produces destructive ^•^OH radicals [57]. These results highlight the specific role of iron metabolism in PSI damage and recovery.

The classical transcription response to abiotic stress, normally strongly induced by HL and involving upregulation of heat shock factors, protein chaperones and cytosolic ascorbate peroxidase (APX2), was significantly under-expressed in *pgr5* (table 2; figure 5*b*). Considering the damaging effect of HL on PSI in *pgr5*, this demonstrates that the classical ‘HL signalling’ cannot be fully induced when PSI activity is inhibited. In comparison, abiotic stress-responsive gene expression in *npq4* was generally slightly (but not significantly) upregulated from WT levels (table 2). Abiotic stress signalling under HL stress is associated with photosynthetic production of H_2_O_2_ [35,36], which derives from superoxide anions (O_2_^•−^) formed at the PSI acceptor side, within the PSI complex and/or in the PQ pool [58–60]. Downregulation of H_2_O_2_ signalling in HL-treated *pgr5* plants reiterates the signalling role of PSI and stromal factor(s) independently of the PQ redox state [48,61], which is similarly over-reduced in both *pgr5* and *npq4* mutants in HL (figure 2*c*; [21]). Under-production of H_2_O_2_ and the altered reduction state of the chloroplast likely impair many redox-regulated signalling pathways that operate through reduction of signalling intermediates, such as TGA transcription factors that regulate detoxification networks [31,62] or nonexpressor of pathogenesis-related 1 (NPR1) required for pathogenesis response (reviewed in [63]).

A large majority of the genes that were downregulated in HL-stressed *pgr5* compared to WT were found to be strongly induced by necrotrophic and herbivorous predators (electronic supplementary material, figure S1), underscoring the importance of both JA and its precursor OPDA, in instigating the response to fungal and insect attacks [64,65]. The transcript profiles of the HL-treated *pgr5* mutant indicate that PSI damage may severely compromise a plant's capacity to deal with stresses of both abiotic and biotic origins. This is likely to have contributed to the high mortality of *pgr5* mutants grown under field conditions [22]. Furthermore, these results reiterate the central role of light-harvesting and photosynthetic electron transport regulation in chloroplast signalling [44,48,49,66], which must be considered in assessments of the fitness and yield of plants with engineered photosynthesis.

## Supplementary Material

Light-induced expression fold change in Col-0

## Supplementary Material

Expression of OPDA-responsive genes in pgr5 and WT in changing light condtions

## Supplementary Material

Expression in public datasets of genes found to be down-regulated in pgr5

## Supplementary Material

High light-induced lipid peroxidation

## Data accessibility

Additional data are provided as electronic supplementary material.
