# Supplementary material for: Interaction between photosynthetic electron transport and chloroplast sinks triggers protection and signalling important for plant productivity
Source: Philos Trans R Soc Lond B Biol Sci. 2017 Aug 14;372(1730):20160390. doi: 10.1098/rstb.2016.0390 (PMC5566885; doi:10.1098/rstb.2016.0390)
Supplement: Expression in public datasets of genes found to be down-regulated in pgr5 [file rstb20160390supp3.pdf]

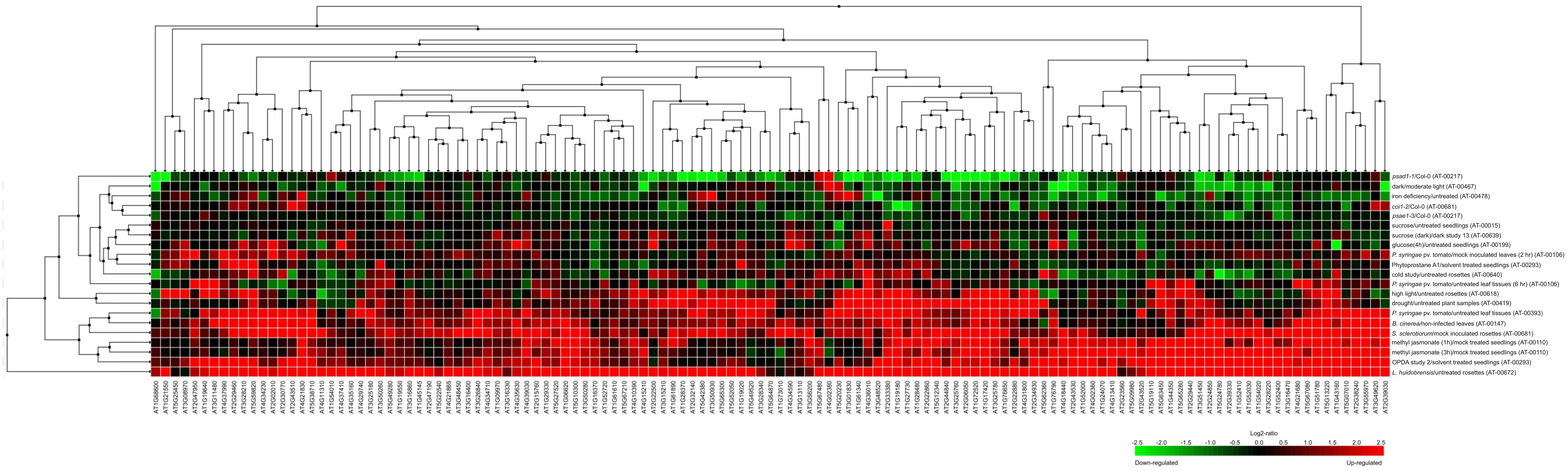

Heatmap showing the expression profiles of attenuated genes in *pgr5* under high light stress and recovery, in publicly-available transcriptomics experiments using Genevestigator (Hruz et al. 2008). Experiment identifiers are given in brackets in the experiment description. Colour indicates the degree of fold change in treatment / control, according to the colour scale shown at the bottom of the page. The expression fold change of a set of 130 genes found to be most strongly down-regulated in *pgr5 / gl1 WT* after 1 hr HL treatment and 1 hr recovery in GL was analysed in selected experimental treatments using Genevestigator. This analysis showed expression of this geneset to be upregulated by methyl jasmonate and 12-oxo-phytodienoic acid (OPDA), which supports the notion that oxylipin signalling is attenuated in *pgr5* mutants after HL exposure. This geneset was strongly induced in plants infected with the herbivore *Liriomyza huidobrensis*, the fungal pathogens *Sclerotinia sclerotiorum* and *Botrytis cinerea*, and the bacterial pathogen *Pseudomonas syringae*. High light and drought stresses also upregulated a large proportion of this geneset. This analysis indicates that the gene expression responses to the above pathogens and stresses may be impaired in *pgr5* mutants after HL exposure. A majority of the geneset was down-regulated in the *psad1* and *psae1* knockout mutants in which PSI function is impaired (Pesaresi et al. 2009), as well in dark-adapted plants and plants grown in iron-deficient conditions. These results suggest that impaired PSI function and/or under-supply of reductants in chloroplast stroma may be responsible for down-regulation of this geneset in the *pgr5* mutant.

- Hruz T, Laule O, Szabo G, Wessendorp F, Bleuler S, Oertle L, Widmayer P, Gruissem W, Zimmermann P. Genevestigator v3: a reference expression database for the meta-analysis of transcriptomes. Advances in bioinformatics. 2008 Jul 8;2008.
- Pesaresi P, Hertle A, Pribil M, Kleine T, Wagner R, Strissel H, Ihnatowicz A, Bonardi V, Scharfenberg M, Schneider A, Pfannschmidt T. Arabidopsis STN7 kinase provides a link between short-and long-term photosynthetic acclimation. The Plant Cell. 2009 Aug 1;21(8):2402-23.
