# Supplementary material for: Interaction between photosynthetic electron transport and chloroplast sinks triggers protection and signalling important for plant productivity
Source: Philos Trans R Soc Lond B Biol Sci. 2017 Aug 14;372(1730):20160390. doi: 10.1098/rstb.2016.0390 (PMC5566885; doi:10.1098/rstb.2016.0390)
Supplement: High light-induced lipid peroxidation [file rstb20160390supp4.pdf]

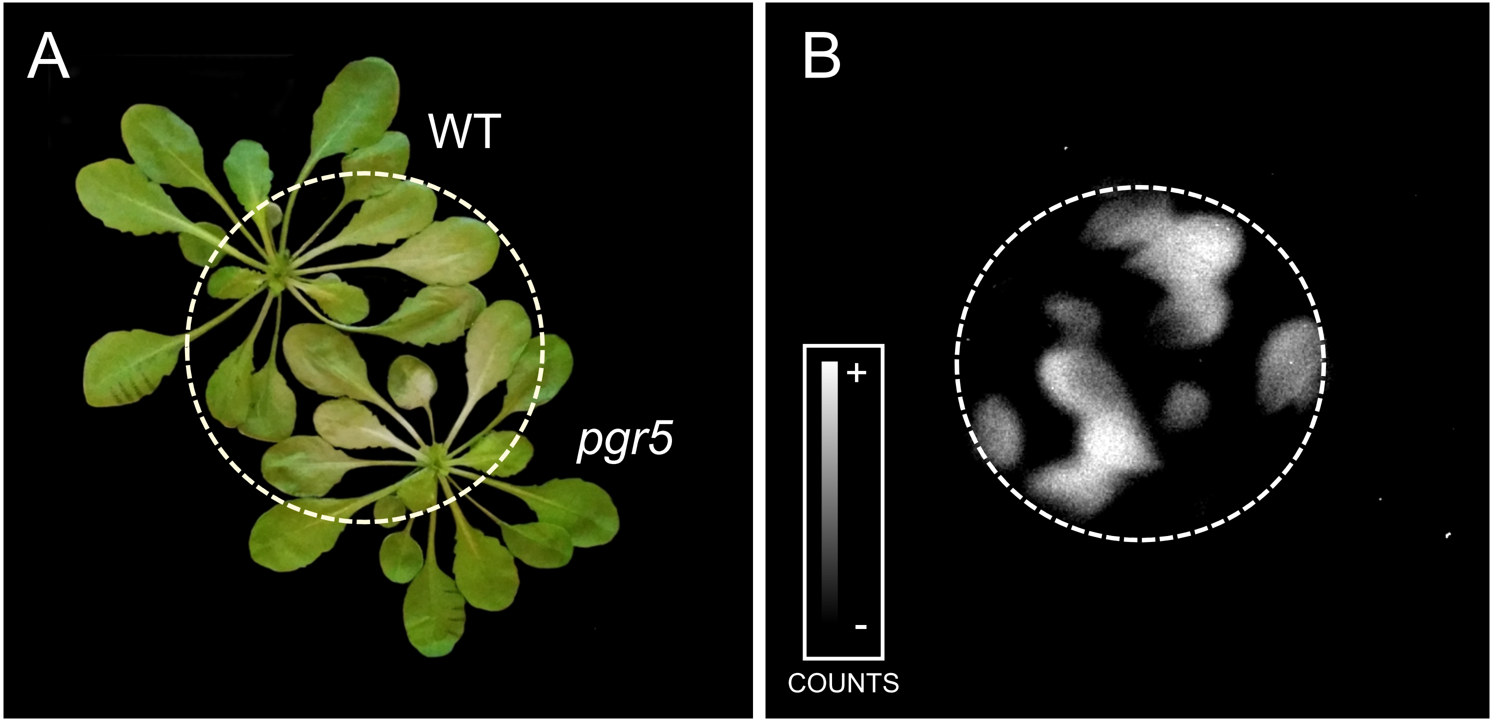

High light-induced lipid peroxidation in WT (*gl1*) and *pgr5* plants.

A. *gl1* (upper) and *pgr5* (lower) rosettes after localised treatment with high light (area within dashed circle)

B. Autoluminescence imaging of plants after light stress. Scale shows relative photon counts.
